# Antioxidant and Tyrosinase-Inhibitory Activities and Biological Bioactivities of Flavonoid Derivatives from *Quercus mongolica* Pollen

**DOI:** 10.3390/molecules30040794

**Published:** 2025-02-08

**Authors:** Yerim Joo, Young Ho Seo, Sangmin Lee, Eunbeen Shin, Sang Won Yeon, Seon Beom Kim, Mi Kyeong Lee

**Affiliations:** 1 Department of Food Science and Technology, Pusan National University, Miryang 50463, Republic of Korea; 2 Institute for Future Earth, Pusan National University, Busan 46421, Republic of Korea; 3 College of Pharmacy, Chungbuk National University, Cheongju 28160, Republic of Korea; 4 Food Tech Innovation Center, Life and Industry Convergence Research Institute, Pusan National University, Miryang 50463, Republic of Korea

**Keywords:** pollen, *Quercus mongolica*, flavonoid glycosides, tyrosinase inhibition, antioxidant

## Abstract

Flavonoids, present in plants as enriched secondary metabolites, prevent various stresses such as temperature fluctuations, acidity, and insect predation, are commonly found in leaves, stems, and flowers, and serve as important bioactive components. In this study, a total of eighteen different flavonoids, including one newly identified flavonoid glycoside, were successfully isolated from the pollen of *Quercus mongolica*. The structure of the novel compound was determined using nuclear magnetic resonance, mass spectrometry, and infrared spectroscopy. Additionally, GC analysis was conducted to determine the sugar moiety in the new compound, confirming the specific type of disaccharide present. The 18 compounds were classified as flavonoid glycosides (**1**–**10**), flavonoids (**11**–**17**), and isoflavone (**18**). All the isolated compounds were evaluated for their tyrosinase inhibitory and antioxidant activities, and their structure–activity relationships (SARs) were also evaluated. Compounds **12** and **16** showed higher tyrosinase inhibitory activities compared to kojic acid as positive control. Compounds **2**, **5**, **8**, **12**, **13**, **14**, and **16** demonstrated potent antioxidant activities. Among these compounds, **5** and **16** showed even higher antioxidant activity than the ascorbic acid. Structure–activity relationship analysis revealed that tyrosinase-inhibitory and antioxidant activities were enhanced in compounds with a hydroxy group of C-3 or C-3′t in flavonoid aglycones compared to their glycosides. These findings indicate that flavonoids and/or extracts from the pollen of *Q. mongolica* are valuable natural resources with applications in the pharmaceutical and cosmetic industries.

## 1. Introduction

Members of the Fagaceae family, which includes *Quercus acutiserrata*, *Quercus aliena* Blume, *Quercus dentata* Thunb, *Quercus mongolica* Fisch. ex Ledeb, *Quercus serrata* Murray, and *Quercus variabilis* Blume, called acorn trees, are representative hardwoods distributed throughout South Korea. Among the acorn trees, *Q. mongolica* is the predominant species in South Korea. Studies of *Q. mongolica* have reported that the essential oils, phenolics (including flavonoids and tannins), polyamines, and their metabolites in these trees have anti-inflammatory, antioxidant, anti-fungal, antimicrobial, and tyrosinase-inhibitory activities due to bioactive components [1,2,3,4,5,6]. However, although there has been a few of phytochemical investigation, research on pollen from *Q. mongolica* remains limited [4,7].

Flowers are pollinated through the transfer of pollen, a process facilitated by wind (anemophily) or insects (entomophily). In entomophily, bees and other insects collect pollen as a food source, transferring it among flowers and thereby enabling pollination. Pollen is a nutrient-dense substance containing well-balanced essential components such as carbohydrates, fats, and proteins, along with a variety of vitamins and minerals [8]. Moreover, it is enriched in multiple metabolites such as phenolics, polyamines, lipids, and alkaloids [4,9,10,11,12,13,14,15,16,17,18,19,20,21,22,23,24,25,26,27]. The biological activities of pollen have been reported to include anti-inflammatory, antioxidant, antibacterial, and anti-fungal effects [28,29,30,31,32].

Flavonoids, the major constituents in plant pollen, are representative antioxidants and are involved in protecting pollen from environmental changes by being distributed on the exine [33,34,35]. Flavonoids in pollen primarily occur as glycosylated forms and have been reported to account for approximately 1–5% of the total extract, depending on the floral source [36,37,38]. Kaempferol and quercetin are well-known examples of flavonoid aglycones, which are flavonoids without any attached sugar moiety; notably, isorhamnetin is also frequently found as an aglycone in bee-collected pollen. In contrast, the glycosides consist of complex carbohydrates with high chemical and structural diversity [10,33,34,35]. Flavonoid glycosides in pollen can act as pollen germination factors by controlling the level of bioactive flavanol [39]. Moreover, flavonoid glycosides in pollen helps prevent cytoplasmic damage by increasing the polarity and enabling their safe storage in the cell vacuole. Consequently, derivatized flavonoids are likely prevalent in plants [40]. Thus, flavonoids have been employed as biomarkers of plant pollens of natural resources by high-performance liquid chromatography (HPLC-UV) for quantitative and qualitative analysis [10,41]. As pollen is derived from a diverse range of plant species, it contains an extensive array of flavonoids and their associated glycosides [2].

Polyamines are among the major constituents of plant pollen and are polycationic molecules containing amino groups [42]. Within plant systems, the predominant polyamines include putrescine, spermidine, and spermine. These compounds are synthesized sequentially through the biosynthetic pathway of arginine [43,44]. Polyamines are involved in various functions related to plant growth, stress, and disease resistance and play important roles in cell growth, survival, and proliferation [42,43,45,46]. The tyrosinase-inhibitory activity of polyamines isolated from acorn pollen has been previously evaluated, with the results suggesting that polyamines in pollen obtained from *Q. mongolica* are candidates for tyrosinase inhibition [4,36].

Tyrosinase, a copper-dependent enzyme found extensively throughout nature, is a critical regulatory factor in the melanocytes’ melanin synthesis pathway [47]. The generation of melanin primarily determines skin pigmentation and serves as a vital protective mechanism against ultraviolet-induced skin damage [48]. However, excessive accumulation of melanin can cause freckles or hyperpigmentation, leading to increased interest in tyrosinase inhibitors within the pharmaceutical and cosmetic industries [49,50]. Antioxidants are commonly employed as food additives to protect products from free-radical-induced oxidative deterioration. Free radicals are molecules characterized by unpaired electrons, rendering them both chemically unstable and exceptionally reactive. Free radicals, such as reactive oxygen species and reactive nitrogen species, induce inflammation by causing cellular damage in humans. Such cellular impairment is linked to the onset of cancer, various cardiovascular conditions—including stroke—and neurodegenerative disorders [51,52]. Flavonoids, a major class of secondary metabolites found in pollen, are well-known antioxidants. Flavonoids can neutralize electrophiles and free radicals through their phenolic hydroxy groups by donating electrons [53]. Therefore, consumption of pollen may help prevent oxidative-stress-induced aging and the onset of chronic diseases [24,28,54,55].

Using theoretical and experimental approaches, we identified flavonoids and polyamines as the primary compounds present in pollen. In previous studies [4,35], polyamines were isolated and purified from *Q. mongolica*. In the present work, bioactive flavonoid compounds were characterized using various spectroscopic methods, and their biological activities—including tyrosinase inhibition and 2,2-diphenyl-1-picrylhydrazyl (DPPH) radical scavenging—were evaluated. Because flavonoids exhibit substantial antioxidant capacity, a structure–activity relationship (SAR) analysis was performed to investigate how molecular configuration influences their biological functions. This approach revealed key correlations between the structural features of flavonoids and their associated biological activities.

## 2. Results and Discussion

### 2.1. Structural Determination of New Compound

**Figure 1:**
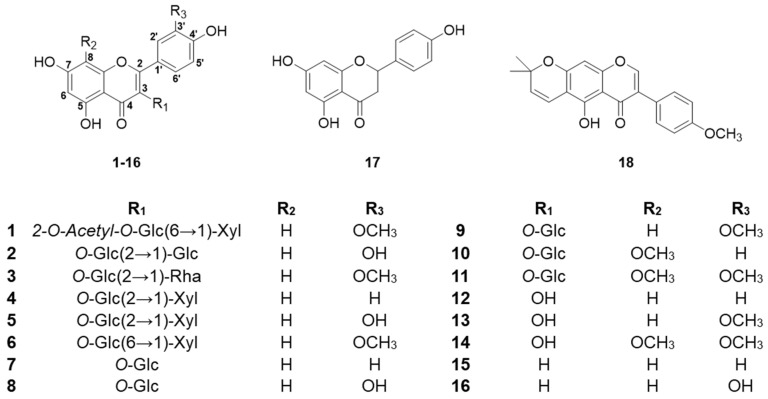
Structures of flavonoid derivatives identified from the acorn pollen of *Q. mongolica*. Abbreviations for carbohydrate units are as follows: Glc for glucopyranoside, Rha for rhamnopyranoside, Xyl for xylopyranoside, and Ara for arabinopyranoside.

**Figure 2:**
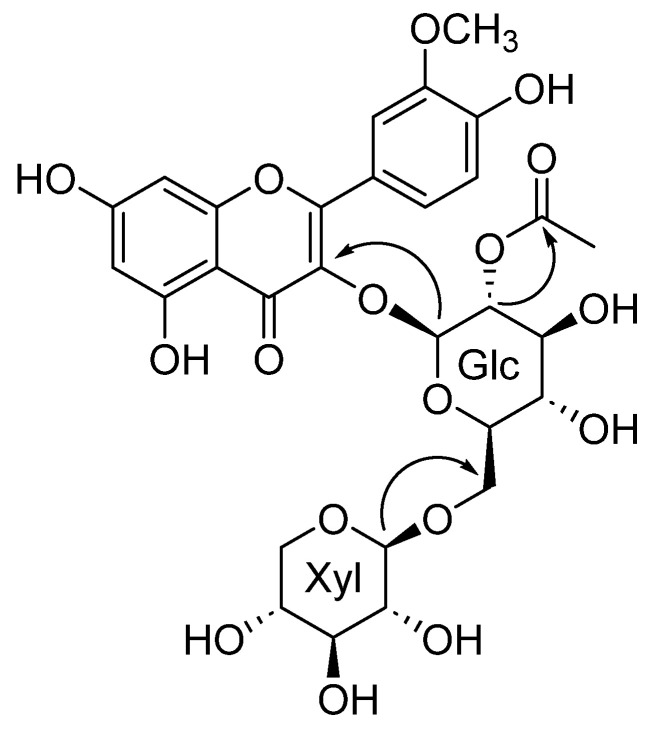
Key HMBC correlations for compound 1. These correlations showed the specific attachment sites of the two carbohydrate groups on the flavonoid aglycone.

Compound **1** was obtained as a white, amorphous powder. Its molecular formula, determined through HRESIMS, was confirmed as C_29_H_32_O_17_ based on the [M+H]^+^ ion at *m*/*z* 653.1691 (calculated *m*/*z* 653.1718). The FT-IR spectrum of compound **1** identified functional groups, as evidenced by characteristic absorption bands for hydroxyl groups near 3263 cm^−1^, a ketone group at around 1729 cm^−1^, and olefinic groups at approximately 1651 cm^−1^. The ^1^H and ^13^C NMR chemical shift of compound **1** is shown on Table 1. The ^1^H and ^13^C NMR spectra revealed a typical flavonoid and two carbohydrate peaks. The A ring of the flavonoids exhibited meta-coupled aromatic protons, observed at δ_H_ 6.13 (1H, s, H-6) and δ_H_ 6.35 (1H, s, H-8). In contrast, the B ring was characterized as a 1,3,4-substituted aromatic system, displaying signals at δ_H_ 6.90 (1H, d, *J* = 8.4 Hz), δ_H_ 7.60 (1H, dd, *J* = 2.1, 8.4 Hz), and δ_H_ 7.96 (1H, d, *J* = 2.1 Hz). The C ring was fully substituted at all three positions. The position of the methoxy group (OCH_3_-3′) has been established based on the observed OCH_3_-3′/C-3′ cross-peak in the ^1^H-^13^C HMBC correlation. Furthermore, the location of the quaternary carbon (C-3′ and 4′) has been determined from the H-6′/C-4′ cross-peak identified in the ^1^H-^13^C HMBC correlation. This evidence of ^1^H-^13^C HMBC cross peaks confirms that the aglycone structure is isorhamnetin. Carbohydrate components were identified through ¹H and ¹³C NMR spectroscopy. For *β*-_D_-glucose, characteristic proton signals were observed at δ_H_ 5.58 (1H, d, *J* = 8.0 Hz, H-1′′), 4.99 (1H, dd, *J* = 8.0, 9.4 Hz, H-2′′), 3.67 (1H, dd, *J* = 9.0, 9.4 Hz, H-3′′), 3.41 (1H, dd, *J* = 9.0, 9.9 Hz, H-4′′), 3.53 (1H, m, H-5′′), 3.58 (1H, dd, *J* = 6.0, 11.4 Hz, H-6′′a), and 3.96 (1H, dd, *J* = 2.0, 11.4 Hz, H-6′′b), supported by corresponding carbon resonances at δ_C_ 100.81 (C-1′′), 75.64 (C-2′′), 75.64 (C-3′′), 71.53 (C-4′′), 77.49 (C-5′′), and 69.38 (C-6′′). Additionally, the *β*-_D_-xylose moiety exhibited distinctive proton signals at δ_H_ 4.06 (1H, d, *J* = 7.5 Hz, H-1′′′), 3.05 (1H, dd, *J* = 7.5, 9.1 Hz, H-2′′′), 3.15 (1H, t, *J* = 9.0 Hz, H-3′′′), 3.37 (1H, m, H-4′′′), 2.90 (1H, dd, *J* = 10.0, 11.3 Hz, H-6a′′′), and 3.65 (1H, dd, *J* = 5.3, 11.3 Hz, H-6b′′′), along with corresponding carbon signals at δ_C_ 105.20 (C-1′′′), 74.72 (C-2′′′), 77.49 (C-3′′′), 71.02 (C-4′′′), and 66.50 (C-5′′′). The ^1^H-^13^C HMBC analysis confirmed the specific glycosidic linkage between the carbohydrate units and the aglycone. It demonstrated that *β*-_D_-glucose was attached to the flavonoid core at the C-3 position (H-1′′/C-3). Additionally, a (6→1)-*β* linkage between *β*-_D_-xylose and *β*-_D_-glucose was established based on the H-1′′′/C-6′′ cross-peak observed in the ^1^H-^13^C HMBC spectrum. The correlation of δ_C_ 172.45 (carbonyl) with the *β*-_D_-glucose proton at H-2′′ (δ_H_ 4.99) indicated that an acetyl group was substituted at C-2′′ Figure 1 and Figure 2. Upon hydrolysis, compound **1** confirmed _D_-glucose and _D_-xylose, subsequently identified as their alditol acetates by GC-MS. These findings collectively led to the characterization of compound **1** as a novel flavonoid glycoside, as shown in Figure 1, named mongolinodoside A.

### 2.2. Structural Identification of Known Compounds

The 17 known flavonoid compounds, quercetin 3-sophoroside (**2**) [56], calendoflavoside (**3**) [57], leucoside (**4**) [58], quercetin 3-sambubioside (**5**) [59], Isorhamnetin 3-*O*-*β*-_D_-xylopyranosyl (1→6)-*β*-_D_-glucopyranoside (**6**) [60], astragalin (**7**) [61], isoquercetin (**8**) [62], isorhamnetin 3-glucoside (**9**) [63], 8-methoxykaempferol 3-glucoside (**10**) [64], limocitrin 3-glucoside (**11**) [65], kaempferol (**12**) [66], isorhamnetin (**13**) [67], limocitrin (**14**) [68], apigenin (**15**) [69], luteolin (**16**) [70], naringenin (**17**) [71], and 4′-methylalpinumisoflavone (**18**) [72], were determined from the spectroscopic data in comparison with reference literature.

### 2.3. Monosaccharide Composition Analysis of New Compound

**Figure 3:**
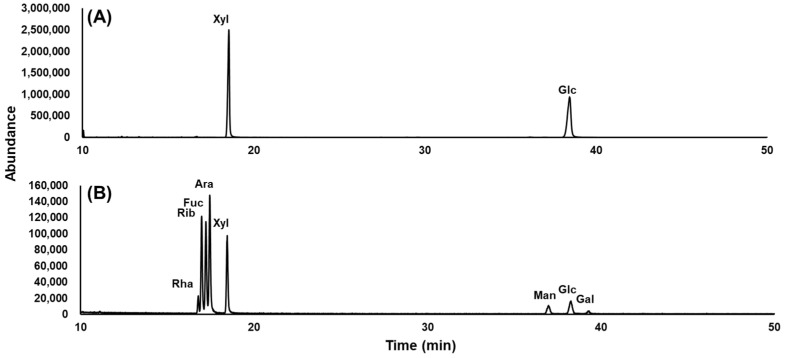
GC-MS chromatograms of (**A**) the glycosidic composition of compound **1** and (**B**) monosaccharide standards. The peaks are annotated as follows: Ara for arabinose, Fuc for fucose, Gal for galactose, Glu for glucose, Rha for rhamnose, Rib for ribose, Man for mannose, and Xyl for xylose.

The monosaccharide profile of the novel flavonoid was analyzed using GC-MS, with the hydrolysis products derivatized as alditol acetates. The total ion chromatogram of the glycosidic composition of the novel flavonoid and eight monosaccharide standards (arabinose, fucose, galactose, glucose, mannose, rhamnose, ribose, and xylose) are shown in Figure 3. The monosaccharide profile of the novel flavonoid was determined by analyzing its retention time compared to monosaccharide reference standards. The GC chromatogram showed two peaks that were identified as _D_-glucose and _D_-xylose.

### 2.4. Tyrosinase-Inhibitory Activity of Isolated Compounds

Tyrosinase is a crucial enzyme in melanin synthesis, participating as an oxidase in the initial steps of melanin formation. The inhibition of tyrosinase activity of compounds **1**–**18**, derived from the pollen of *Q. mongolica*, was assessed using mushroom tyrosinase. The results of these inhibitory activity assessments are shown in Table 2 and Supplementary Materials: Figure S1. Among the isolated compounds, compound **12** exhibited highly potent inhibition, showing over 70% tyrosinase inhibition at a concentration of 100 μM and an IC_50_ value of 20.9 µM. This inhibitory effect was stronger than that of the positive control, kojic acid, which had an IC_50_ of 36.4 µM. Compound **16** showed over 50% tyrosinase inhibition, with an IC_50_ value of 38.8 µM. Structure–activity relationship (SAR) analysis indicated that compound **12**, containing a hydroxyl group of C-3, showed significant inhibitory activity compared to compound **15**. Compound **16** with a hydroxy group at C-3′ showed higher activity than compound **15**. When comparing compound **12** with compounds **4** and **7**, and compound **16** with compounds **2** and **8**, aglycones **12** and **16** demonstrated higher activity. Among the compounds **1**–**18** isolated from the pollen of *Q. mongolica*, those containing a hydroxyl group position at the C-3 or C-3′ and aglycone structures exhibited stronger tyrosinase-inhibitory activity compared to that in glycosides.

### 2.5. Antioxidant Activity of Isolated Compounds

The antioxidant properties of compounds **1**–**18** isolated from the pollen of *Q. mongolica* were assessed through DPPH assay. The free-radical-scavenging activity of DPPH assay results are shown in Figure S1 and Table 2. Compounds **2**, **5**, **8**, **12**, **13**, **14**, and **16** exhibited over 90% scavenging activity at a concentration of 100 µM. Compounds **2** (IC_50_ of 34.3 µM), **5** (IC_50_ of 18.4 µM), **8** (IC_50_ of 28.5 µM), **13** (IC_50_ of 25.5 µM), and **16** (IC_50_ of 9.7 µM) showed IC_50_ comparable to the positive control, ascorbic acid (IC_50_ of 28.5 µM). Notably, compounds **5** and **16** exhibited the highest antioxidant activities, which were higher than that of ascorbic acid. SAR analysis demonstrated that compound **12**, with the C-3 of hydroxyl group, exhibited stronger radical-scavenging activity compared to compound **15**. Similarly, compound **16**, containing at C-3′ a hydroxyl group, showed higher activity than compound **15**. When comparing compound **12** with compounds **4** and **7**, compound **13** with compounds **1**, **3**, and **6**, and compound **11** with **14**, the aglycones (**12**, **13**, and **14**) displayed superior antioxidant activity. Based on SAR (structure–activity relationship) analysis, flavonoid derivatives isolated from the pollen of *Q. mongolica* with a hydroxyl group at C-3 or C-3′ and aglycone structures exhibited higher antioxidant activity compared to their glycoside counterparts.

## 3. Experimental Sections

### 3.1. General Experimental Procedures

The analysis of all isolated compounds was carried out using the following instruments: UV spectral data were acquired with a Waters 2996 Photodiode Array Detector (Waters Corporation, Milford, MA, USA), while IR spectra were obtained by using a Nicolet iS10 FT-IR spectrometer (Thermo Fisher Scientific, Waltham, MA, USA). NMR spectra were obtained from a Bruker AVANCE III 900 MHz (Bruker, Billerica, MA, USA) equipped with a ^1^H-(^13^C/^15^N)Z-G cryogenic probe, using CD_3_OD as the solvent. High-resolution electrospray ionization mass spectrometry (HRESI-MS) analysis was conducted using a Bruker maXis spectrometer (Bruker, Billerica, MA, USA). Open-column chromatography utilized silica gel (Merck Millipore, Billerica, MA, USA) and Sephadex LH-20 (Sigma Aldrich, St. Louis, MO, USA). Medium-pressure liquid chromatography (MPLC) was employed in a Biotage Isolera flash column system (Biotage, Uppsala, Sweden), while semi-prep HPLC data were obtained with Waters 515 pumps (Waters Corporation, Milford, MA, USA) and a 2995 PDA system (Waters Corporation, Milford, MA, USA). Finally, GC-MS analysis was conducted on an Agilent 5977C GC/MSD instrument (Agilent, Santa Clara, CA, USA).

### 3.2. Plant Materials

The pollen samples used in this research were obtained from the Rural Development Administration located in Jeonbuk, Republic of Korea. These pollen samples were dried at 40 °C and kept at −20 °C for storage until analysis. To determine the species, Dr. In Pho Hong performed identification using colorimetric methods and electron microscopy to verify the taxonomy.

### 3.3. Extraction and Isolation of Flavonoids from Pollen of Q. Mongolica

The collected pollen of *Q. mongolica* (15.0 kg) was extracted with 80% methanol, yielding 2.2 kg of 80% MeOH extracts. The pollen extracts of *Q. mongolica* were dissolved in water and partitioned stepwise using *n*-hexane, dichloromethane (CH_2_Cl_2_), ethyl acetate (EtOAc), and *n*-butanol (*n*-BuOH). Using semi-prep HPLC under a MeOH:H_2_O gradient (40:60 → 100:0) as the eluent, compounds **2** (0.41 mg), **3** (5.96 mg), **6** (1.74 mg), and **7** (3.53 mg) were obtained from the total extract. The EtOAc fraction (108.5 g) was chromatographed to silica gel with a CH_2_Cl_2_:MeOH step gradient (100:0 → 0:100) as the mobile phase, resulting in 21 fractions (APEA01–APEA21). The APEA01 fraction was further fractionated over the Sephadex LH-20 eluted with CH_2_Cl_2_:MeOH (2:1), yielding seven sub-fractions (APEA01A–APEA01G). From the APEA01F fraction, compound **18** (0.47 mg) was obtained as a single molecule. The APEA06 fraction underwent purification using Sephadex LH-20 under the CH_2_Cl_2_:MeOH (2:1) solvent system, resulting in seven distinct sub-fractions labeled as APEA06A through APEA06G. Compound **14** (14.66 mg) was obtained from the APEA06F fraction using semi-prep HPLC with MeOH:H_2_O (40:60) as the eluent. The APEA07 fraction underwent purification using Sephadex LH-20 under the CH_2_Cl_2_:MeOH (2:1) solvent system. This process generated 11 sub-fractions, labeled APEA07A through APEA07K. From the APEA07H fraction, compound **17** (1.92 mg) was isolated using semi-prep HPLC with MeOH:H_2_O (60:40) as the eluent.

The *n*-BuOH fraction (600.0 g, APB) was suspended in water, and the insoluble residue (APBC) was collected as a clump (66.3 g). The APBC fraction was chromatographed to silica gel with a CH_2_Cl_2_:MeOH gradient (100:0 → 0:100), resulting in 18 sub-fractions (APBC01–APBC18). The APBC11 fraction underwent MPLC on a reversed-phase silica column with a MeOH:H_2_O gradient (10:90 → 100:0), yielding five fractions (APBC11A–APBC11E). The APBC11C was fractionated on a Sephadex LH-20 column with 100% MeOH, producing four fractions (APBC11C01–APBC11C04). Compounds **10** (5.86 mg) and **11** (1.93 mg) were isolated from the APBC11C03 fraction using semi-prep HPLC with MeOH:H_2_O (32:68) as the eluent. The APBC13 fraction was separated by MPLC under the reversed-phase silica column with a MeOH:H_2_O gradient (10:90 → 100:0), producing six fractions (APBC13A–APBC13F). The APBC13A was fractionated on a Sephadex LH-20 column with 100% MeOH, yielding four sub-fractions (APBC13A01–APBC13A04). Compound **4** (1.79 mg) was isolated from the APBC13A03 fraction using semi-prep HPLC with MeOH:H_2_O (37:63) under isocratic conditions. The APBC13B fraction underwent MPLC with Sephadex LH-20 and 100% MeOH as the eluent, producing three fractions (APBC13B01–APBC13B03). Compounds **5** (1.83 mg) and **8** (0.5 mg) were obtained from the APBC13B02 fraction using semi-prep HPLC with MeOH:H_2_O (45:55) as the eluent under isocratic conditions. The *n*-BuOH fraction (600.0 g, APB) was chromatographed in the XAD column with a MeOH:H_2_O gradient (100:0 → 0:100), resulting in five fractions (APB01–APB05). The APB04 fraction was fractionated using MPLC on a silica column with a CH_2_Cl_2_:MeOH gradient (100:0 → 0:100), yielding 12 sub-fractions (APB04A–APB04L). The APB04C fraction was fractionated over the Sephadex LH-20 column chromatography with MeOH, resulting in 11 sub-fractions (APB04C01–APB04C11). Compounds **9** (0.05 mg) and **15** (0.94 mg) were obtained from the APB04C05 fraction using semi-prep HPLC with MeOH:H_2_O (55:45). Similarly, compounds **12** (0.65 mg), **13** (12.12 mg), and **16** (2.79 mg) were isolated from the APB04C10 fraction using semi-prep HPLC under isocratic conditions with MeOH:H_2_O (55:45) as the eluent. Compound **1** (47.5 mg) was obtained from the APB04H fraction via MPLC using a CH_2_Cl_2_:MeOH gradient.

### 3.4. New Compound

White amorphous powder; [α]^D^ −33.98° (c 4.75, MeOH); UV (MeOH) λ_max_ 253.6 (1.81) nm; IR_max_ 3263 (O-H), 1729 (C=O), 1651 (C=C) cm^−1^; ^1^H NMR (CD_3_OD, 900 MHz) and ^13^C NMR (CD_3_OD, 225 MHz); HRESI-TOFMS 653.1691 [M+H]^+^(calcd. for C_29_H_33_O_17_, 653.1718).

### 3.5. Monosaccharide Composition Analysis

#### 3.5.1. Monosaccharide Hydrolysis and GC-MS Analysis for Composition Assay

In the hydrolysis step, 300 µg of each sample was placed in 4 mL vials containing 2.0 M trifluoroacetic acid and heated at 120 °C for 90 min. The vials were then cooled and trifluoroacetic acid was removed. Methanol was added to remove residual trifluoroacetic acid, followed by evaporation. During the reduction step, a solution of 1.0 M aqueous ammonia diluted with dimethyl sulfoxide was prepared. Each sample was treated with this solution and incubated at 40 °C for 90 min, with vortexing every 30 min. The reaction was quenched with glacial acetic acid. For the acetylation step, 1-methylimidazole and anhydrous acetic anhydride were added to the samples, which were incubated at 40 °C for 30 min to complete the reaction. The purification step involved transferring the reaction mixture to a new vial containing distilled water and dichloromethane. The organic layer was partitioned by centrifugation, and the washing step with distilled water was repeated four times. For GC-MS analysis, the organic layer was dried and evaporated in dichloromethane. The acetate derivatives were analyzed using GC-MS, employing monosaccharides, as shown in Figure 3, as reference standards.

#### 3.5.2. GC-MS Analytical Conditions

The analytical conditions for the GC-MS used an Agilent 8890 gas chromatograph equipped with a 5977C mass selective detector (Agilent Technologies). Separation was performed on an HP-5ms ultra-inert column (30 m × 0.25 mm × 0.25 µm). The temperature program for the oven started at 80 °C, held for 2 min, and after that increased at a rate of 30 °C/min to 170 °C. A gradual rise at 0.1 °C/min brought the temperature to 175 °C, followed by a rapid increase of 30 °C/min until 280 °C, where it remained stable for 20 min. The inlet temperature was maintained at 250 °C, and the carrier gas of helium flowed at 1.0 mL/min. The ion source temperature remained constant at 250 °C, while the mass spectrometer operated under electron ionization conditions at 70 eV. The mass range scanned between 40 and 550 *m*/*z*. For compound identification, the National Institute of Standards and Technology (NIST) library was utilized.

### 3.6. Tyrosinase Inhibition Assay and Antioxidant Assay

#### 3.6.1. Tyrosinase Inhibition Assay

Evaluation of tyrosinase-inhibitory activity was conducted using mushroom tyrosinase (Sigma-Aldrich). For each test, 10 mM of each compound isolated from the pollen of *Q. mongolica* (1 µL) was evaluated. A 50 µL portion of 1 mM tyrosine (Sigma-Aldrich) dissolved in phosphate-buffered saline was introduced into each well, followed by a 5 min incubation period. Mushroom tyrosinase was prepared to a final concentration of 0.1 U/mL in phosphate-buffered saline, and 49 µL of the prepared enzyme solution was added to the wells, resulting in a compound concentration of 0.1 mM. The mixture underwent a 15 min incubation, after which the production of dopachrome was evaluated by recording the absorbance at 490 nm using a Dynatech MR 500 plate reader (Dynatech, Charlottesville, VA, USA). Kojic acid (Sigma-Aldrich) was used as a positive control. The percentage inhibition for each sample was determined at concentrations of 100 µg/mL. The IC_50_ value was calculated as the concentration of the compound required to achieve 50% inhibition of tyrosinase activity.

#### 3.6.2. Antioxidant Assay

The radical scavenging activity of DPPH (Sigma-Aldrich) was hired for the antioxidant activity. For each assay, 10 mM of each compound isolated from the pollen of *Q. mongolica* (1 µL) was evaluated, followed by 49 µL of MeOH in each well. Subsequently, 50 µL of 0.3 mM DPPH solution (Sigma-Aldrich) was added. The final concentration was tested to 0.1 mM. The mixture was incubated for 10 min, and antioxidant activity was evaluated by measuring UV at 550 nm using a Dynatech MR 500 plate reader. The percentage inhibition was calculated for each sample at concentrations of 100 µg/mL. The IC_50_ value was tested with different concentrations of samples that showed 50% of the antioxidant activity.

## 4. Conclusions

In this study, eighteen compounds, including flavonoids, flavonoid glycosides, and isoflavones, were isolated and purified from the pollen of *Q. mongolica* through chromatographic techniques. Their structures were elucidated by analyzing spectroscopic data and comparing it with previously reported information in the literature. A novel flavonoid glycoside, mongolinodoside A, was also identified. Its structure consists of *β*-_D_-glucose and *β*-_D_-xylose linked via a (6→1)-*β* bond.

The tyrosinase-inhibitory and radical-scavenging activities of the isolated flavonoids were evaluated. Compounds **12** and **16** demonstrated tyrosinase inhibition levels comparable to kojic acid, while compounds **2**, **5**, **8**, **12**, **13**, **14**, and **16** exhibited antioxidant activities comparable to ascorbic acid. Among these, compounds **5** and **16** showed significantly strong antioxidant activity. Compounds with a hydroxy group at C-3 or C-3′ and aglycones exhibited enhanced both tyrosinase-inhibitory and antioxidant activities. Based on this study, flavonoids isolated from the pollen of *Q. mongolica* are a promising natural commercial source for use in the pharmaceutical and cosmetic industries.

## Figures and Tables

**Table 1:** The ^1^H and ^13^C NMR chemical shift of compound **1**.

Compound 1 ^a^									
Position	δ_H_, m (J in Hz)		δ_C_	Type	Position	δ_H_, m (J in Hz)		δ_C_	Type
1			-		1′′	5.58, d	(8.0)	100.80	CH
2			158.44	C	2′′	4.99, dd	(9.4, 8.0)	75.64	CH
3			134.71	C	3′′	3.67, dd	(9.4, 9.0)	75.64	CH
4			178.85	C	4′′	3.41, dd	(9.9, 9.0)	71.53	CH
5			162.76	C	5′′	3.53, m		77.49	CH
6	6.13, s		99.80	CH	6′′	3.96, dd	(11.4, 1.9)	69.38	CH_2_
7			165.74	C		3.58, dd	(11.4, 6.0)		
8	6.35, s		94.90	CH	1′′′	4.06, d	(7.5)	105.02	CH
9			158.18	C	2′′′	3.05, dd	(9.1, 7.5)	74.72	CH
10			105.84	C	3′′′	3.15, t	(8.9)	77.49	CH
1′			123.10	C	4′′′	3.37, m		71.02	CH
2′	7.96, d	(2.1)	114.50	CH	5′′′	3.65, dd	(11.3, 5.3)	66.50	CH_2_
3′			148.32	CH		2.90, dd	(11.3, 10.0)		
4′			150.64	C	OCH_3_-3′	3.99, s		56.84	CH_3_
5′	6.90, d	(8.4)	116.00	C	OAc-2′′	2.16, s		21.20	CH_3_
6′	7.60, dd	(8.4, 2.1)	123.60	CH	2′′-COO^-^	-		172.45	C

^a^ = ^1^H and ^13^C NMR data were recorded at 900 MHz and 225 MHz in CD_3_OD. m = multiplicity.

**Table 2:** IC_50_ values of compounds **1**–**18** on tyrosinase-inhibitory and antioxidant activities.

Compounds	Tyrosinase-Inhibitory Activity	Antioxidant Activity
	IC_50_ Values (µM)	
**1**	>100	>100
**2**	>100	34.3
**3**	>100	>100
**4**	>100	>100
**5**	>100	18.4
**6**	>100	>100
**7**	>100	>100
**8**	>100	28.5
**9**	>100	>100
**10**	>100	>100
**11**	>100	>100
**12**	20.9	42.4
**13**	>100	25.5
**14**	>100	52.3
**15**	>100	>100
**16**	38.8	9.7
**17**	>100	>100
**18**	>100	>100
PC ^a^	36.4	28.5

^a^ = Positive control: kojic acid and ascorbic acid.

## Data Availability

The original contributions presented in this study are included in the article/Supplementary Materials. Further inquiries can be directed to the corresponding authors. Additionally, the raw NMR data for the natural products have been deposited in the Harvard Dataverse (dataverse.harvard.edu) and are accessible at DOI: [https://doi.org/10.7910/DVN/MSTJNR].

## Supplementary Materials

The following supporting information can be downloaded at: https://www.mdpi.com/article/10.3390/molecules30040794/s1, Figure S1: Effect of flavonoids on tyrosinase-inhibitory and antioxidant activities. Figure S2: ¹H NMR spectroscopic data of compound **1**; Figure S3: ¹³C NMR spectroscopic data of compound **1**; Figure S4: DEPT-135 spectrum of compound **1**; Figure S5: ¹H-¹H COSY; spectroscopic data of compound **1**; Figure S6: ¹H-¹³C HSQC spectroscopic data of compound **1**; Figure S7: ¹H-¹³C HMBC spectroscopic data of compound **1**; Figure S8: HRESI-TOF data of compound **1**; Figure S9: UV spectrum of compound **1**.

## References

[1] Suh M., Lee D. (1998). Stand Structure and Regeneration of Quercus Mongolica Forests in Korea. For. Ecol. Manag..

[2] Yin J., Kim H.H., Hwang I.H., Kim D.H., Lee M.W. (2019). Anti-Inflammatory Effects of Phenolic Compounds Isolated from *Quercus Mongolica* Fisch. ex Ledeb. on UVB-Irradiated Human Skin Cells. Molecules.

[3] Ishimaru K., Nonaka G.-I., Nishioka I. (1987). Phenolic glucoside gallates from quercus mongolica and q. acutissima. Phytochemistry.

[4] Kim S.B., Liu Q., Ahn J.H., Jo Y.H., Turk A., Hong I.P., Han S.M., Hwang B.Y., Lee M.K. (2018). Polyamine derivatives from the bee pollen of Quercus mongolica with tyrosinase inhibitory activity. Bioorg. Chem..

[5] Omar M., Matsuo Y., Maeda H., Saito Y., Tanaka T. (2013). New ellagitannin and galloyl esters of phenolic glycosides from sapwood of Quercus mongolica var. crispula (Japanese oak). Phytochem. Lett..

[6] Ishimaru K., Ishimatsu M., Nonaka I., Nishioka G.-I., Mihashi K., Iwase Y. (1988). Tannins and related compounds. LXXI. Isolation and characterization of mongolicins A and B, novel flavono-ellagitannins from quercus mongolica var grosseserrata. Chem. Pharm. Bull..

[7] Sen N.B., Vovk I., Kırmızıbekmez H., Guzelmeric E. (2024). Phytochemical and Bioactivity Evaluation of Bee Pollen and Androecia of *Castanea*, *Salix*, and *Quercus* Species. Antioxidants.

[8] Kacemi R., Campos M.G. (2023). Translational Research on Bee Pollen as a Source of Nutrients: A Scoping Review from Bench to Real World. Nutrients.

[9] Rzepecka-Stojko A., Stojko J., Kurek-Górecka A., Górecki M., Kabała-Dzik A., Kubina R., Moździerz A., Buszman E. (2015). Polyphenols from Bee Pollen: Structure, Absorption, Metabolism and Biological Activity. Molecules.

[10] Tomás-Barberán F.A., Tomás-Lorente F., Ferreres F., Garcia-Viguera C. (1989). Flavonoids as biochemical markers of the plant origin of bee pollen. J. Sci. Food Agric..

[11] Zhang H., Liu R., Lu Q. (2020). Separation and Characterization of Phenolamines and Flavonoids from Rape Bee Pollen, and Comparison of Their Antioxidant Activities and Protective Effects Against Oxidative Stress. Molecules.

[12] Kempf M., Heil S., Haßlauer I., Schmidt L., von der Ohe K., Theuring C., Reinhard A., Schreier P., Beuerle T. (2010). Pyrrolizidine alkaloids in pollen and pollen products. Mol. Nutr. Food Res..

[13] Di Paola-Naranjo R.D., Sánchez-Sánchez J., González-Paramás A.M., Rivas-Gonzalo J.C. (2004). Liquid chromatographic–mass spectrometric analysis of anthocyanin composition of dark blue bee pollen from Echium plantagineum. J. Chromatogr. A.

[14] Rodríguez-Pólit C., Gonzalez-Pastor R., Heredia-Moya J., Carrera-Pacheco S.E., Castillo-Solis F., Vallejo-Imbaquingo R., Barba-Ostria C., Guamán L.P. (2023). Chemical Properties and Biological Activity of Bee Pollen. Molecules.

[15] Calderón-Martínez P., Yam-Puc A., Ramón-Sierra J., Hernández-Bolio G., Hernández-Núñez E., Zamora-Bustillos R., Ortiz-Vázquez E. (2024). Antioxidant and Antibacterial Properties of Ethanolic Pot-Pollen Extracts of *Melipona beecheii* and Determination of the Major Components by GC-MS. Chem. Biodivers..

[16] Kacemi R., Campos M.G. (2023). Bee Pollen as a Source of Pharmaceuticals: Where Are We Now?. Pollen Chemistry & Biotechnology.

[17] Capparelli S., Pieracci Y., Coppola F., Marchioni I., Sagona S., Felicioli A., Pistelli L., Pistelli L. (2023). The colors of Tuscan bee pollen: Phytochemical profile and antioxidant activity. Nat. Prod. Res..

[18] Seraglio S.K.T., Brugnerotto P., Deolindo C.T.P., Blainski E., Dortzbach D., Santana B.d.O., Hoff R.B., Gonzaga L.V., Costa A.C.O. (2024). LC–MS/MS analysis of pyrrolizidine alkaloids in bee bread and commercial pollen from Brazil. Eur. Food Res. Technol..

[19] Kostić A.Ž., Gercek Y.C., Bayram N.E. (2023). Phenolic Acids in Pollen. Pollen Chemistry & Biotechnology.

[20] Kalaba M., Tešić Ž., Blagojević S. (2023). Flavonoids in Pollen. Pollen Chemistry & Biotechnology.

[21] Rivest S., Muralidhar M., Forrest J.R.K. (2024). Pollen chemical and mechanical defences restrict host-plant use by bees. Proc. R. Soc. B Biol. Sci..

[22] Jiang F., Li M., Huang L., Wang H., Bai Z., Niu L., Zhang Y. (2024). Metabolite Profiling and Biological Activity Assessment of *Paeonia ostii* Anthers and Pollen Using UPLC-QTOF-MS. Int. J. Mol. Sci..

[23] Kostić A.Ž., Kilibarda S. (2023). Lipids in Pollen. Pollen Chemistry & Biotechnology.

[24] Kacemi R., Campos M.G. (2025). Bee Pollen Phytochemicals and Nutrients as Unequaled Pool of Epigenetic Regulators: Implications for Age-Related Diseases. Foods.

[25] Bernal J., Valverde S., Fuente-Ballesteros A., Martín-Gómez B., Ares A.M. (2023). Other Bioactive Constituents of Pollen. Pollen Chemistry & Biotechnology.

[26] Zhang H., Lu Q., Liu R. (2021). Widely targeted metabolomics analysis reveals the effect of fermentation on the chemical composition of bee pollen. Food Chem..

[27] Rivest S., Forrest J.R.K. (2020). Defence Compounds in Pollen: Why Do They Occur and How Do They Affect the Ecology and Evolution of Bees?. New Phytol..

[28] Denisow B., Denisow-Pietrzyk M. (2016). Biological and therapeutic properties of bee pollen: A review. J. Sci. Food Agric..

[29] Komosinska-Vassev K., Olczyk P., Kaźmierczak J., Mencner L., Olczyk K. (2015). Bee Pollen: Chemical Composition and Therapeutic Application. Evid.-Based Complement. Altern. Med..

[30] Filannino P., Di Cagno R., Vincentini O., Pinto D., Polo A., Maialetti F., Porrelli A., Gobbetti M. (2021). Nutrients Bioaccessibility and Anti-inflammatory Features of Fermented Bee Pollen: A Comprehensive Investigation. Front. Microbiol..

[31] Fatrcová-Šramková K., Nôžková J., Kačániová M., Máriássyová M., Rovná K., Stričík M. (2013). Antioxidant and antimicrobial properties of monofloral bee pollen. J. Environ. Sci. Health Part B.

[32] LeBlanc B.W., Davis O.K., Boue S., DeLucca A., Deeby T. (2009). Antioxidant activity of Sonoran Desert bee pollen. Food Chem..

[33] Ceska O., Styles E. (1984). Flavonoids from Zea mays pollen. Phytochemistry.

[34] Strack D., Meurer B., Wray V., Grotjahn L., Austenfeld F., Wiermann R. (1984). Quercetin 3-glucosylgalactoside from pollen of *Corylus avellana*. Phytochemistry.

[35] Meurer B., Wray V., Grotjahn L., Wiermann R., Strack D. (1986). Hydroxycinnamic acid spermidine amides from pollen of *Corylus avellana* L.. Phytochemistry.

[36] Kim S.B., Jo Y.H., Liu Q., Ahn J.H., Hong I.P., Han S.M., Hwang B.Y., Lee M.K. (2015). Optimization of Extraction Condition of Bee Pollen Using Response Surface Methodology: Correlation between Anti-Melanogenesis, Antioxidant Activity, and Phenolic Content. Molecules.

[37] Mărgăoan R., Stranț M., Varadi A., Topal E., Yücel B., Cornea-Cipcigan M., Campos M.G., Vodnar D.C. (2019). Bee Collected Pollen and Bee Bread: Bioactive Constituents and Health Benefits. Antioxidants.

[38] Mărghitaş L.A., Stanciu O.G., Dezmirean D.S., Bobiş O., Popescu O., Bogdanov S., Campos M.G. (2009). In vitro antioxidant capacity of honeybee-collected pollen of selected floral origin harvested from Romania. Food Chem..

[39] Taylor L.P., Strenge D., Miller K.D. (1998). The Role of Glycosylation in Flavonol-Induced Pollen Germination. Adv. Exp. Med. Biol..

[40] Qiao J., Feng Z., Zhang Y., Xiao X., Dong J., Haubruge E., Zhang H. (2022). Phenolamide and flavonoid glycoside profiles of 20 types of monofloral bee pollen. Food Chem..

[41] Campos M., Markham K.R., Mitchell K.A., da Cunha A.P. (1997). An Approach to the Characterization of Bee Pollens via Their Flavonoid/Phenolic Profiles. Phytochem. Anal..

[42] Tiburcio A.F., Altabella T., Bitrián M., Alcázar R. (2014). The roles of polyamines during the lifespan of plants: From development to stress. Planta.

[43] Kusano T., Berberich T., Tateda C., Takahashi Y. (2008). Polyamines: Essential factors for growth and survival. Planta.

[44] Minois N., Carmona-Gutierrez D., Madeo F. (2011). Polyamines in aging and disease. Aging.

[45] Minocha R., Majumdar R., Minocha S.C. (2014). Polyamines and abiotic stress in plants: A complex relationship1. Front. Plant Sci..

[46] Gill S.S., Tuteja N. (2010). Polyamines and abiotic stress tolerance in plants. Plant Signal. Behav..

[47] Olivares C., Solano F. (2009). New insights into the active site structure and catalytic mechanism of tyrosinase and its related proteins. Pigment. Cell Melanoma Res..

[48] Zolghadri S., Bahrami A., Hassan Khan M.T., Munoz-Munoz J., Garcia-Molina F., Garcia-Canovas F., Saboury A.A. (2019). A comprehensive review on tyrosinase inhibitors. J. Enzym. Inhib. Med. Chem..

[49] Chang T.-S. (2009). An Updated Review of Tyrosinase Inhibitors. Int. J. Mol. Sci..

[50] Odonbayar B., Murata T., Batkhuu J., Yasunaga K., Goto R., Sasaki K. (2016). Antioxidant Flavonols and Phenolic Compounds from *Atraphaxis frutescens* and Their Inhibitory Activities against Insect Phenoloxidase and Mushroom Tyrosinase. J. Nat. Prod..

[51] Carocho M., Ferreira I.C.F.R. (2013). A review on antioxidants, prooxidants and related controversy: Natural and synthetic compounds, screening and analysis methodologies and future perspectives. Food Chem. Toxicol..

[52] Biswas S., Das R., Banerjee E.R. (2017). Role of free radicals in human inflammatory diseases. AIMS Biophys..

[53] Campos M.G.R., Frigerio C., Lopes J., Bogdanov S. (2010). What Is the Future of Bee-Pollen?. J. Apiproduct Apimedical Sci..

[54] Di Renzo L., Gualtieri P., Frank G., Cianci R., Caldarelli M., Leggeri G., Raffaelli G., Pizzocaro E., Cirillo M., De Lorenzo A. (2024). Exploring the Exposome Spectrum: Unveiling Endogenous and Exogenous Factors in Non-Communicable Chronic Diseases. Diseases.

[55] Noor S.N.M., Musa M., Azlina A., Gan S.H., Thirumulu K.P. (2024). Polyphenols in bee products and prevention of cell senescence. BioMedicine.

[56] Cui E.-J., Song N.-Y., Shrestha S., Chung I.-S., Kim J.-Y., Jeong T.-S., Baek N.-I. (2012). Flavonoid glycosides from cowpea seeds (Vigna sinensis K.) inhibit LDL oxidation. Food Sci. Biotechnol..

[57] Peng W., Li Y., Zhu C., Han X., Yu B. (2005). Synthesis of tamarixetin and isorhamnetin 3-O-neohesperidoside. Carbohydr. Res..

[58] Saito N., Toki K., Honda T., Tatsuzawa F. (2012). ChemInform Abstract: Floral Pigments Isolated from the Sky-Blue Flowers of Oxypetalum caeruleum. ChemInform.

[59] Webby R.F. (1991). A flavonol triglycoside from Actinidia arguta var. Giraldii. Phytochemistry.

[60] Tomás-Lorente F., Garcia-Grau M.M., Nieto J.L., Tomás-Barberán F.A. (1992). Flavonoids from Cistus ladanifer bee pollen. Phytochemistry.

[61] Kazuma K., Noda N., Suzuki M. (2003). Malonylated flavonol glycosides from the petals of Clitoria ternatea. Phytochemistry.

[62] Han J.-T., Bang M.-H., Chun O.-K., Kim D.-O., Lee C.-Y., Baek N.-I. (2004). Flavonol glycosides from the aerial parts ofAceriphyllum rossii and their antioxidant activities. Arch. Pharmacal. Res..

[63] Lee Y.S., Lee H.S., Shin K.H., Kim B.-K., Lee S. (2004). Constituents of the Halophyte Salicornia herbacea. Arch. Pharmacal. Res..

[64] Rayyan S., Fossen T., Nateland H.S., Andersen Ø.M. (2005). Isolation and Identification of Flavonoids, Including Flavone Rotamers, from the Herbal Drug ‘Crataegi Folium Cum Flore’ (Hawthorn). Phytochem. Anal..

[65] Olszewska M.A., Roj J.M. (2011). Phenolic constituents of the inflorescences of *Sorbus torminalis* (L.) Crantz. Phytochem. Lett..

[66] Ding H., Lin H., Teng C., Wu Y. (2000). Phytochemical and Pharmacological Studies on Chinese *Paeonia* Species. J. Chin. Chem. Soc..

[67] Lee H.-J., Lee H.-J., Lee E.-O., Ko S.-G., Bae H.-S., Kim C.-H., Ahn K.-S., Lu J., Kim S.-H. (2008). Mitochondria-Cytochrome C-Caspase-9 Cascade Mediates Isorhamnetin-Induced Apoptosis. Cancer Lett..

[68] Horie T., Tsukayama M., Kawamura Y., Seno M., Yamamoto S. (1988). Studies of the Selective *O*-Alkylation and Dealkylation of Flavonoids. XI. A New Convenient Method for Synthesizing 3,5,7-Trihydroxy-8-Methoxyflavones from 7-Hydroxy-3,5,8-Trimethoxyflavones. Bull. Chem. Soc. Jpn..

[69] Miyazawa M., Hisama M. (2003). Antimutagenic Activity of Flavonoids from *Chrysanthemum morifolium*. Biosci. Biotechnol. Biochem..

[70] Park Y., Moon B.-H., Lee E., Lee Y., Yoon Y., Ahn J.-H., Lim Y. (2007). 1H and 13C-NMR Data of Hydroxyflavone Derivatives. Magn. Reson. Chem..

[71] Khan M.K., Rakotomanomana N., Loonis M., Dangles O. (2010). Chemical Synthesis of Citrus Flavanone Glucuronides. J. Agric. Food Chem..

[72] Han X.H., Hong S.S., Hwang J.S., Jeong S.H., Hwang J.H., Lee M.H., Lee M.K., Lee D., Ro J.S., Hwang B.Y. (2005). Monoamine oxidase inhibitory constituents from the fruits of *Cudrania tricuspidata*. Arch. Pharmacal. Res..

