# Supplementary material for: Antioxidant and Tyrosinase-Inhibitory Activities and Biological Bioactivities of Flavonoid Derivatives from Quercus mongolica Pollen
Source: Molecules. 2025 Feb 8;30(4):794. doi: 10.3390/molecules30040794 (PMC11858624; doi:10.3390/molecules30040794)
Supplement: Supplementary file 1 [file molecules-30-00794-s001.zip › molecules-3440540-supplementary.pdf]

Supplementary Materials

Table of Contents

S1. Effects of flavonoids on tyrosinase inhibitory and antioxidant activities

S2. ^{1}H NMR spectrum of compound **1** in CD_3OD at 900 MHz

S3. ^{13}C NMR spectrum of compound **1** in CD3OD at 225 MHz

S4. DEPT-135 spectrum of compound **1** in CD_3OD at 225 MHz

S5. ^{1}H-^{1}H COSY spectrum of compound **1** in CD_3OD at 900 MHz

S6. ^{1}H-^{13}C DEPT-HSQC spectrum of compound **1** in CD_3OD at 225 MHz

S7. ^{1}H-^{13}C HMBC spectrum of compound **1** in CD_3OD at 225 MHz

S8. HRESIMS spectrum of compound **1**

S9. UV spectrum of compound **1**

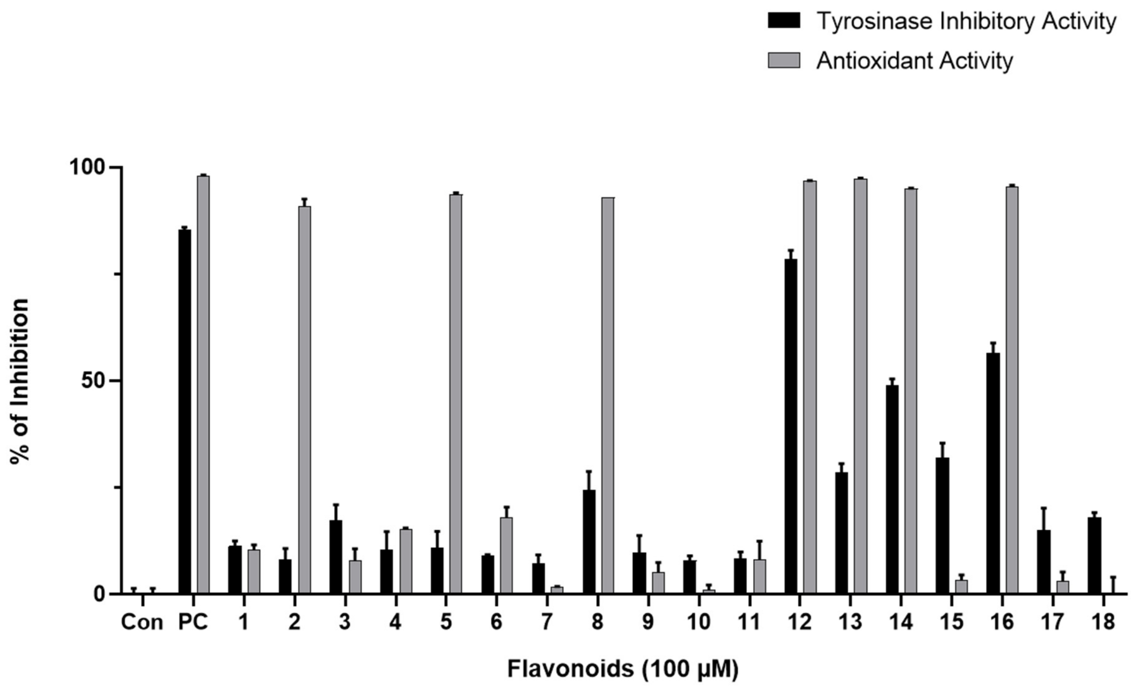

Figure S1. Effects of flavonoids on tyrosinase inhibitory and antioxidant activities

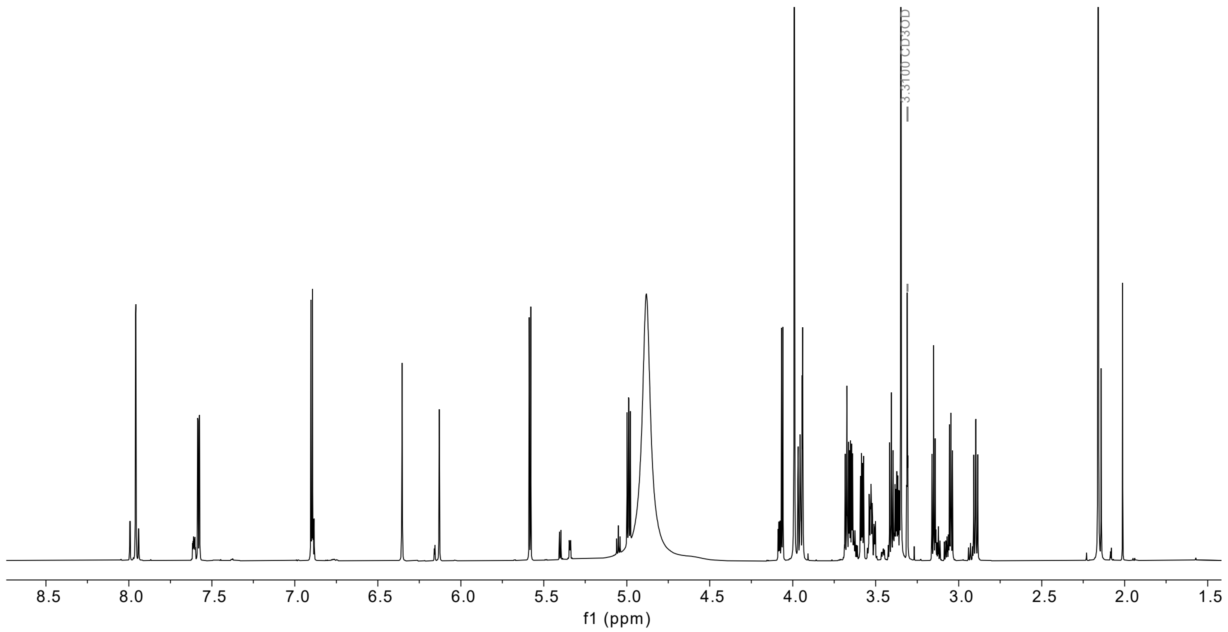

Figure S2. ^{1}H NMR spectrum of compound **1** in CD3OD at 900 MHz

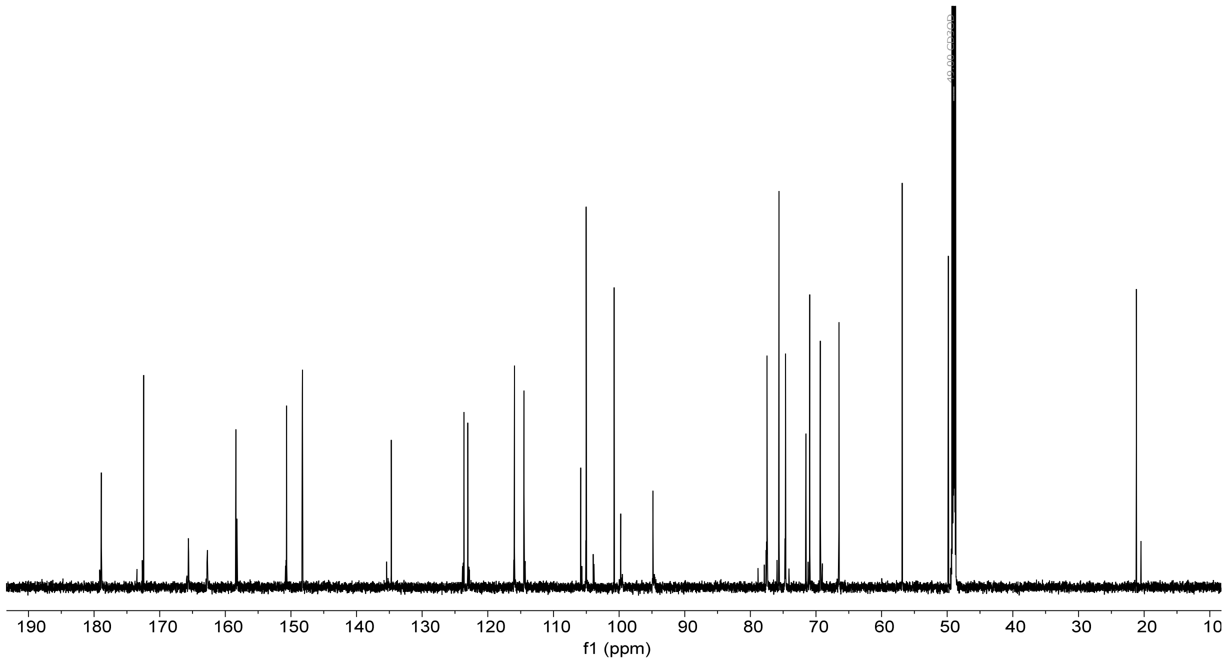

Figure S3. ^{13}C NMR spectrum of compound **1** in CD3OD at 900 MHz

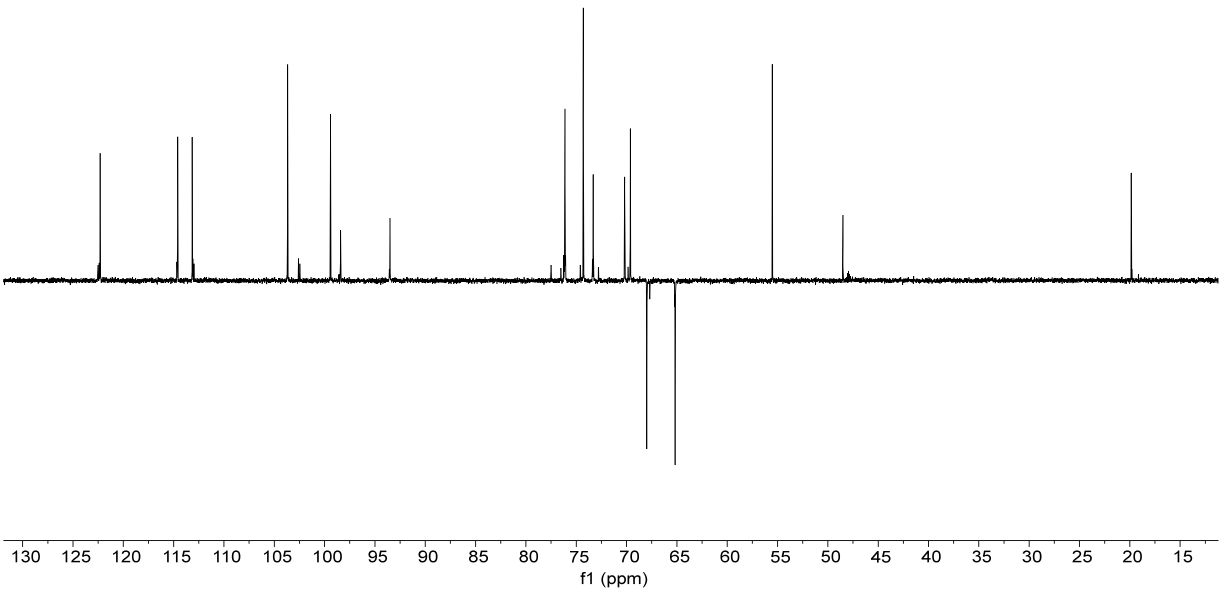

Figure S4. DEPT 135 spectrum of compound **1** in CD_3OD at 225 MHz

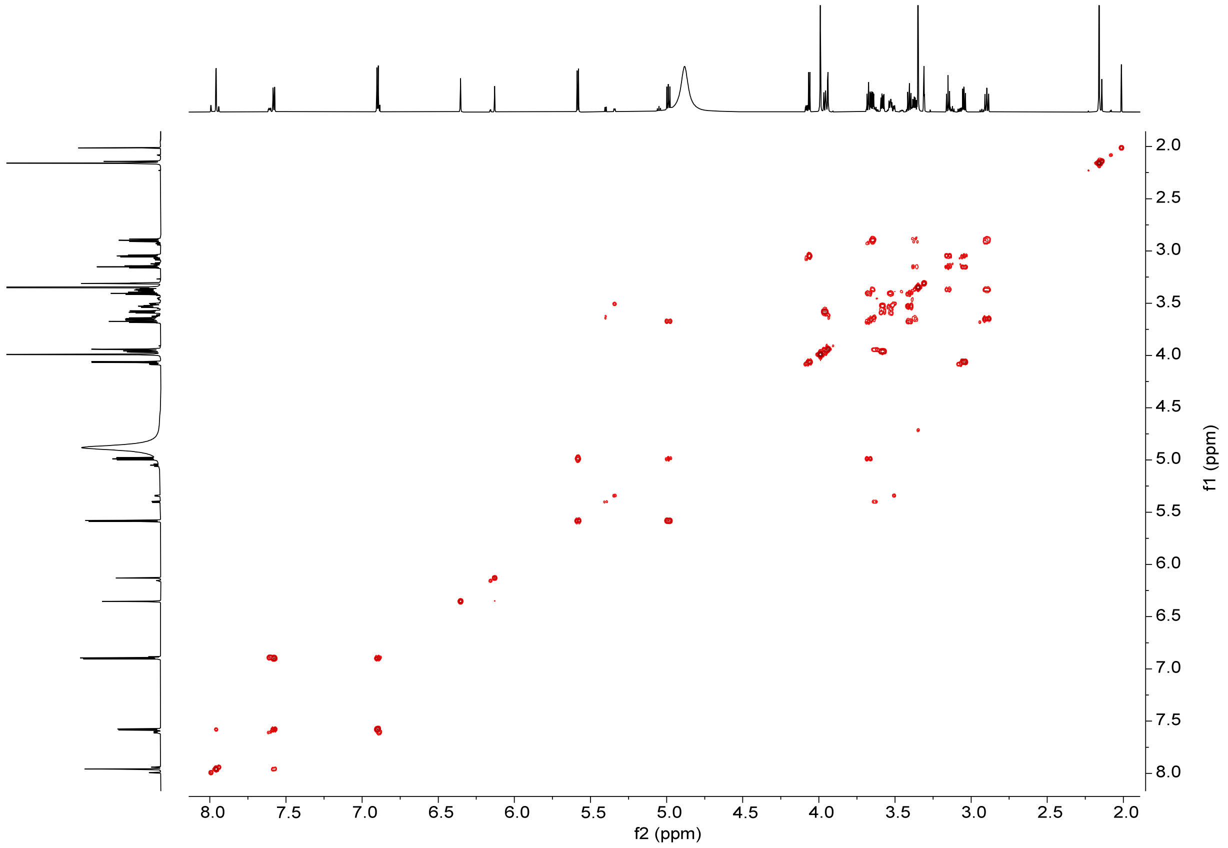

Figure S5. ^{1}H-^{1}H COSY spectrum of compound **1** in CD_3OD at 900 MHz

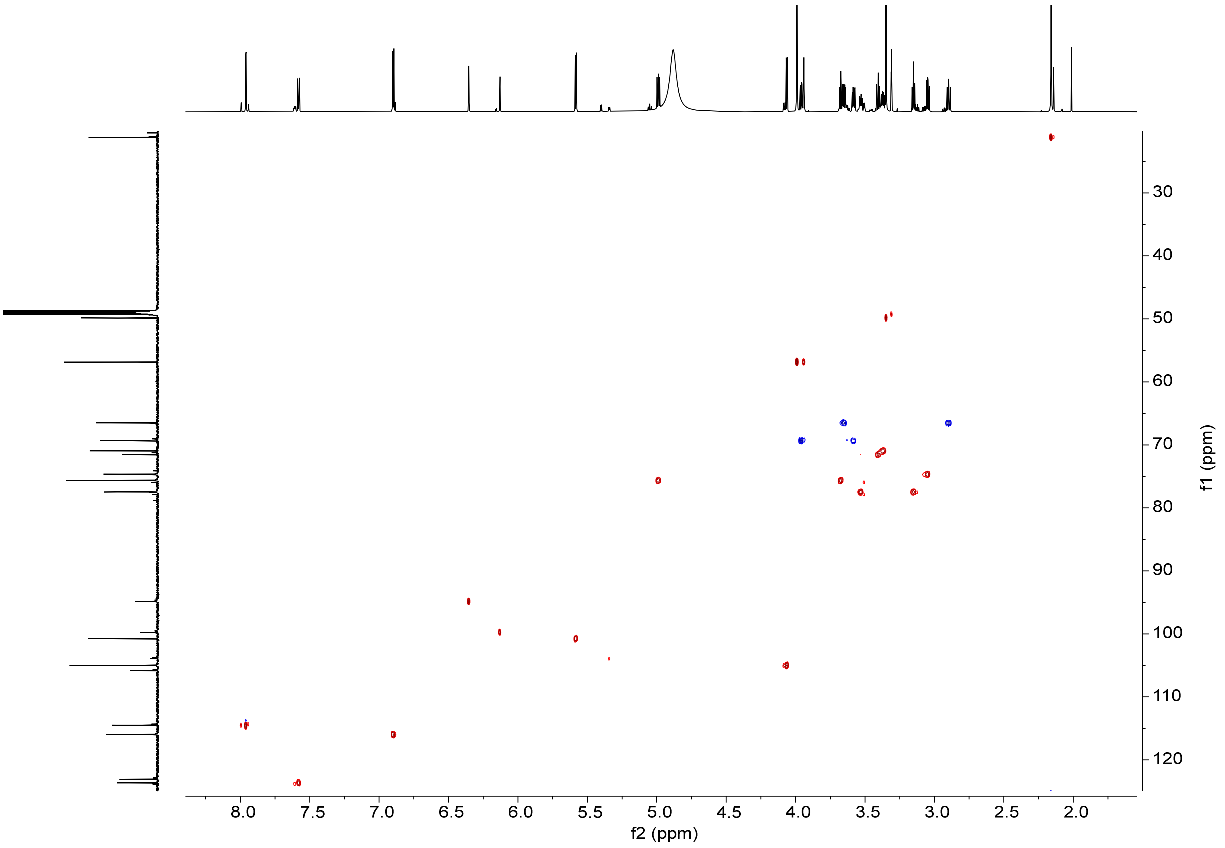

Figure S6. ^{1}H-^{13}C DEPT-HSQC spectrum of compound **1** in CD_3OD at 225 MHz

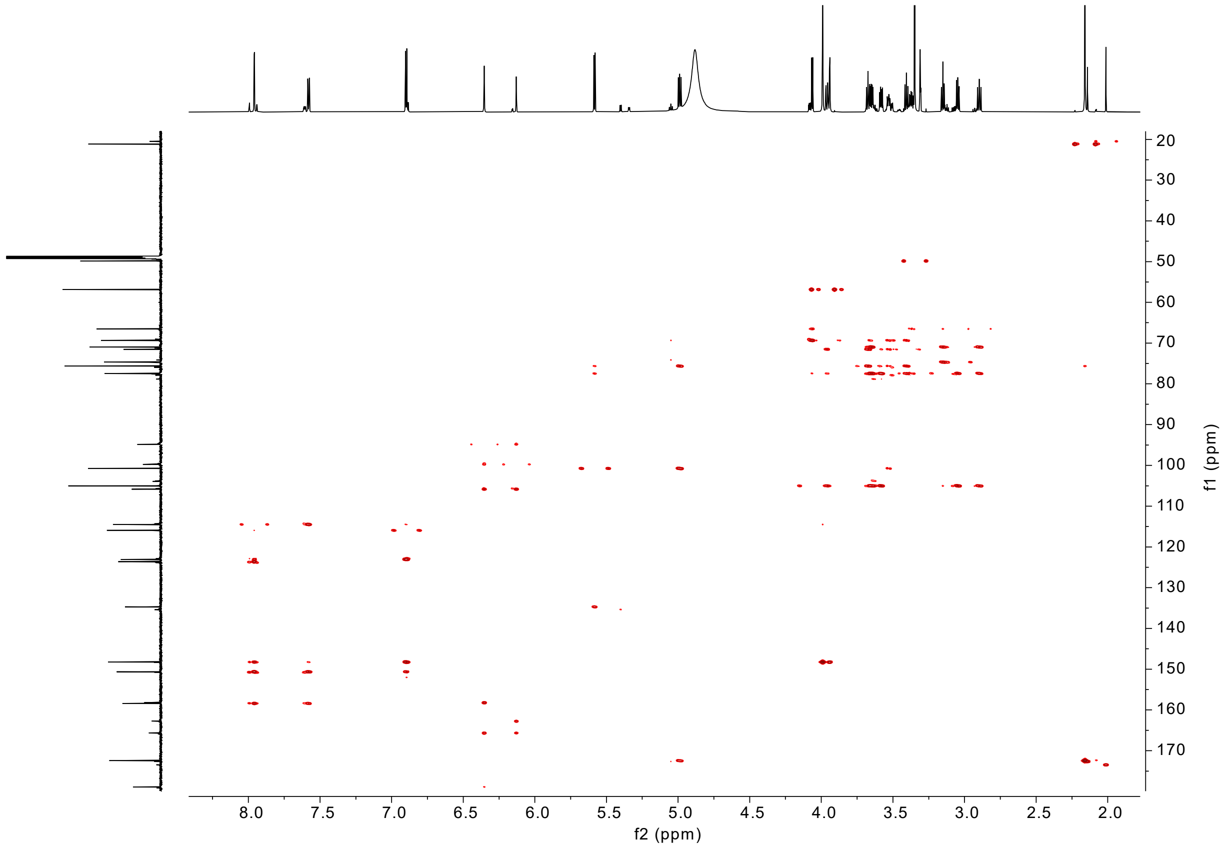

Figure S7. ^{1}H-^{13}C HMBC spectrum of compound **1** in CD_3OD at 225 MHz

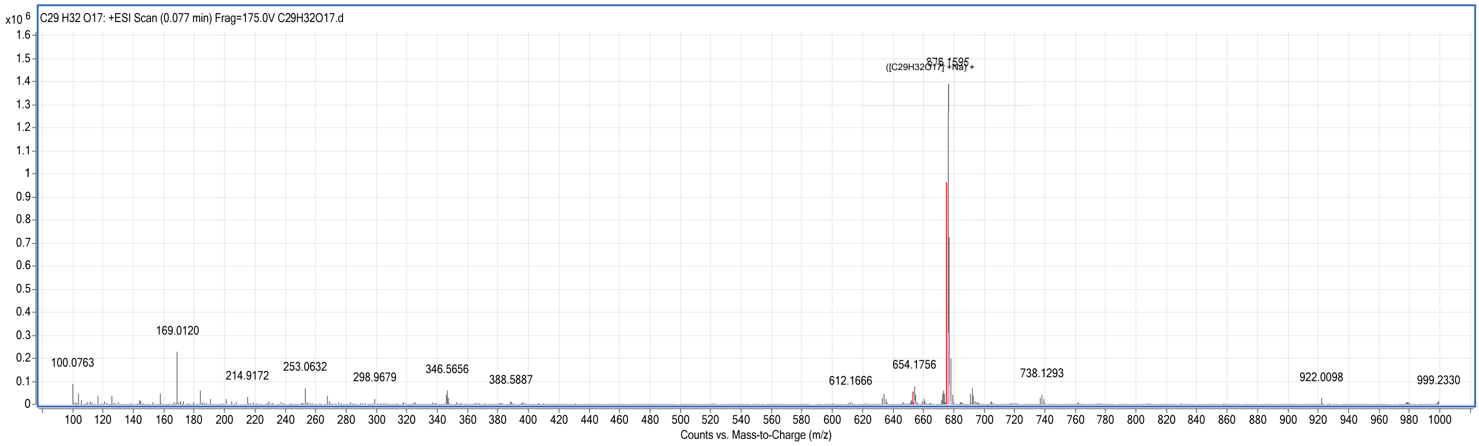

Figure S8. HRESIMS spectrum of compound **1**

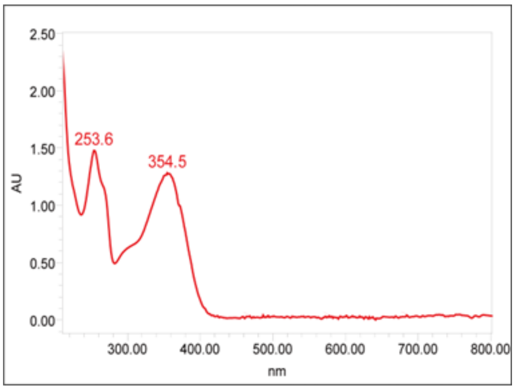

Figure S9. UV spectrum of compound **1**
